# A review of the impacts of degradation threats on soil properties in the UK

**DOI:** 10.1111/sum.12212

**Published:** 2015-10-12

**Authors:** A. S. Gregory, K. Ritz, S. P. McGrath, J. N. Quinton, K. W. T. Goulding, R. J. A. Jones, J. A. Harris, R. Bol, P. Wallace, E. S. Pilgrim, A. P. Whitmore

**Affiliations:** ^1^ Department of Sustainable Soils and Grassland SystemsRothamsted ResearchHarpendenHertfordshireAL5 2JQUK; ^2^ Division of Agricultural and Environmental SciencesUniversity of NottinghamSutton Bonington CampusLoughboroughLeicestershireLE12 5RDUK; ^3^ Lancaster Environment CentreLancaster UniversityLancasterLA1 4YQUK; ^4^ School of Energy, Environment and AgrifoodCranfield UniversityCranfieldBedfordshireMK43 0ALUK; ^5^ Terrestrial Biogeochemistry GroupInstitute of Bio‐ and Geosciences IBG‐3: AgrosphereForschungszentrum Jülich GmbHJülich52425Germany; ^6^ Phil Wallace LtdWestlandMartlesham HeathIpswichSuffolkIP5 3SUUK; ^7^ University of ExeterAmory BuildingRennes DriveExeterEX4 4RJUK

**Keywords:** Soil erosion, soil organic matter, soil contamination, soil compaction, soil functions, climate change

## Abstract

National governments are becoming increasingly aware of the importance of their soil resources and are shaping strategies accordingly. Implicit in any such strategy is that degradation threats and their potential effect on important soil properties and functions are defined and understood. In this paper, we aimed to review the principal degradation threats on important soil properties in the UK, seeking quantitative data where possible. Soil erosion results in the removal of important topsoil and, with it, nutrients, C and porosity. A decline in soil organic matter principally affects soil biological and microbiological properties, but also impacts on soil physical properties because of the link with soil structure. Soil contamination affects soil chemical properties, affecting nutrient availability and degrading microbial properties, whilst soil compaction degrades the soil pore network. Soil sealing removes the link between the soil and most of the ‘spheres’, significantly affecting hydrological and microbial functions, and soils on re‐developed brownfield sites are typically degraded in most soil properties. Having synthesized the literature on the impact on soil properties, we discuss potential subsequent impacts on the important soil functions, including food and fibre production, storage of water and C, support for biodiversity, and protection of cultural and archaeological heritage. Looking forward, we suggest a twin approach of field‐based monitoring supported by controlled laboratory experimentation to improve our mechanistic understanding of soils. This would enable us to better predict future impacts of degradation processes, including climate change, on soil properties and functions so that we may manage soil resources sustainably.

## Introduction

National governments are becoming increasingly aware of the importance of their soil resources and have begun to shape policies to recognize this. In the UK, for example, there have been soil strategies and policy documents in England (HMG, 2009b, 2011b), Scotland (Dobbie *et al*., 2011) and Wales (WAG, 2006). All have a similar central aim, namely the ambition to manage soils sustainably and tackle potential degradation threats so that their ability to provide essential services is protected or enhanced. Soil degradation in England has been estimated to have cost the economy at least £150M–£250M each year (HMG, 2011b).

Implicit in any such strategy is that the principal degradation threats and their effect on important soil properties are defined and understood. If this can be achieved then, by extension, we may better understand the likely impact on soil functions – the essential services that we rely on soils to perform – and we can begin to define policies to protect them. Evidence‐gathering is an initial stage in the process of developing and refining policies (HMG, 2011a).

In this paper, we aimed to identify and review the principal degradation threats on important soil properties in the UK, seeking quantitative data where possible. We summarize the main findings and discuss the likely subsequent impacts on soil functions and also the potential impacts of predicted changes in climate in the UK.

## Methodology

### Degradation threats

In examining which degradation threats were important in the context of the UK, we consulted the report of the European Environmental Assessment of Soil for Monitoring (ENVASSO) project (Huber *et al*., 2008), which aimed to establish a soil monitoring system in support of a proposed European Union‐wide Soil Framework Directive (EC, 2006). In the report of Huber *et al*. (2008), nine key threats to soil were identified: soil erosion, decline in soil organic matter (SOM), soil contamination, soil sealing, soil compaction, decline in soil biodiversity, soil salinization, landslides and desertification. In addition, the report identified three further ‘cross‐cutting issues’ that exist as threats to soil: climate change, land use change and brownfield development. Similar threats to soils have been identified in the UK previously (e.g. SG, 2009; HMG, 2011b).

From this list, we focused on soil erosion, decline in SOM, soil contamination, soil compaction, soil sealing and brownfield development, as we believe these to be of the greatest relevance to soils in the UK. It is our view that the decline in soil biodiversity does not exist as a specific independent threat to soil, but that instead it arises as a result of some of the other threats identified. Similarly, land‐use change imparts some of the other threats identified on the soil. Therefore, these two threats were covered in the context of the other threats. We review soil salinization and landslides only briefly, as we consider these to be less important, and extremely localized in the UK. Desertification is not a threat currently to UK soils and is not reviewed. Finally, we discuss climate change separately in terms of what the likely impacts might be on degradation threats and soil properties. The degradation threats considered and brief definitions are given in Table 1.

**Table 1:** The eight degradation threats identified as important for soils in the UK. The descriptions are those of Huber *et al*. (2008), unless stated otherwise

Degradation threat	Brief description
Soil erosion	The accelerated loss of soil as a result of anthropogenic activity in excess of accepted rates of natural soil formation
Decline in soil organic matter	The negative imbalance between the build‐up of soil organic matter and rates of decomposition, leading to an overall decline in soil organic matter content and/or quality
Soil contamination	The presence of a substance or agent in the soil as a result of human activity emitted from moving sources, from sources with a large area or from many sources (diffuse) (ISO, 2006), or where intensive industrial activities, inadequate waste disposal, mining, military activities or accidents introduce excessive amounts of contaminants (local)
Soil compaction	The densification and distortion of soil by which total and air‐filled porosity are reduced
Soil sealing	The destruction or covering of soil by buildings, constructions and layers or other bodies of artificial material which may be very slowly permeable to water (Burghardt *et al*., 2004)
Brownfield development	The further development of developed land previously used for commercial or industrial purposes, including restoration of green after‐uses
Soil salinization	The excessive increase of water‐soluble salts in soil through natural processes and human interventions
Landslides	The movement of a mass of rock, debris, artificial fill or soil down a slope under the force of gravity (Cruden & Varnes, 1996)
Climate change	The large‐scale, long‐term shift in the planet's weather patterns or average temperatures (Met Office, 2015)

### Literature review

A literature review of peer‐reviewed publications was conducted using the web‐based ISI Web of Knowledge ^SM^ search engine (Thomson Reuter, New York, USA), supplemented by other relevant published evidence (e.g. reports). We took each degradation threat as a search term in turn with ‘soil’ and ‘UK’ and sought data, predominantly from the 2000 to 2015 period, on the impacts on important soil physical, biological and chemical properties in the UK. In particular, we focused on general properties likely to be of direct relevance for the ability of the soil to carry out its principal functions, including food and fibre production, water and air filtration, and biodiversity support. We synthesized the most relevant information in our review below.

### The impacts of degradation threats on soil properties in the UK

#### Soil erosion

Although soil erosion can be natural, current concerns relate to accelerated erosion, where the rate has increased significantly through human activities and greatly exceeds current estimates of soil formation (0.3–1.4 Mg/ha/yr in Europe) (Verheijen *et al*., 2009). Most erosion is by water, with light‐textured soils particularly susceptible (Quinton & Catt, 2004), although wind erosion has been observed on arable soils in eastern England, albeit it is very difficult to quantify (Bullock, 1987), and peaty soils can erode at daily rates of up to 5.6 kg/ha when dry (Foulds & Warburton, 2007).

Mean annual erosion rates in England measured directly or interpreted from aerial photography on mostly arable sites are typically up to 10 Mg/ha, but can be as high as 66 Mg/ha, whilst annual tillage erosion in the UK can reach 5 Mg/ha (van Oost *et al*., 2009). Under grassland, 52% of the 399 upland sites in England and Wales surveyed by McHugh *et al*. (2002) in 1999 had suffered erosion and an estimated 89 000 m^3^ of soil had been lost over an unspecified time, <1% of which was deposited in the same field site. Improved lowland grasslands typically have lower annual rates of erosion, ranging from 0.5 to 1.2 Mg/ha (Whitmore *et al*., 2004; Bilotta *et al*., 2010).

Structural damage of the soil surface, caused by raindrops, running water and deposited material, reduces infiltration and increases surface runoff (Dexter, 1997; Whitmore *et al*., 2004; Pilgrim *et al*., 2010). The direct loss of soil C by water erosion (Lal, 2003; van Oost *et al*., 2007) has been estimated to be 0.20–0.76 Tg annually in England and Wales (Quinton *et al*., 2006). Plant nutrients can be lost in eroded topsoil (Pimentel *et al*., 1995; Quinton *et al*., 2001; Palmer & Smith, 2013), although few published studies have examined N and P losses in detail (Kronvang, 1990; Chambers *et al*., 2000).

#### Decline in soil organic matter

In common with other temperate‐zone countries, there has been a decline in SOM in some soils in the UK over a long period, predominantly as a result of changes in land use. Historically land has changed from native woodland to grazing land, and then on to annual arable systems. Since the 1940s, the area covered by permanent grassland has reduced and in arable systems, there has been a reduction in the use of manures and an increase in cultivation depth (Bellamy *et al*., 2005). These have all served to both reduce organic inputs and increase decomposition of existing SOM. Topsoil horizons (up to 0.30 m depth) are those most affected by SOM decline as the topsoil is the main zone affected by land use, although there is increased awareness of potential impacts of land use on subsoil SOM (e.g. Gregory *et al*., 2014). SOM contents under arable systems are much smaller than those under grass and woodland in the UK (Chapman *et al*., 2013; Gregory *et al*., 2014). The SOM changes as the soil adjusts to a new equilibrium of plant inputs, which can take several decades (Jenkinson *et al*., 2008; Johnston *et al*., 2009). Occasionally land is taken out of arable cultivation and SOM can recover, though at a much slower rate (Johnston, 1986; Poulton *et al*., 2003; Smith *et al*., 2007). There is conflicting evidence as to whether SOM and C stocks have declined significantly in the UK over the last 30 yrs where there has been no change in land use (Bellamy *et al*., 2005; Chapman *et al*., 2013; Reynolds *et al*., 2013), and the current estimate of the total soil organic C stock down to 1 m depth in the whole of the UK is 5260 Tg (Bradley *et al*., 2005; Gregory *et al*., 2014). Instead, there is much evidence as to the effects of SOM decline on soil properties, particularly physical properties.

Specific decreases in aggregate stability of 10–40% with a 1% decrease in SOM content have been recorded in the UK and similar locations (Williams, 1971; Tisdall & Oades, 1980, 1982; Riley *et al*., 2008), as well as a decrease in the resilience of soil to physical stresses, such as compaction (Watts & Dexter, 1997; Gregory *et al*., 2009). This can lead to an undesirable domination of coarser clods (Riley *et al*., 2008), a decrease in friability (Watts & Dexter, 1998) and a reduction in porosity (Riley *et al*., 2008). SOM decline can reduce soil water retention (Kibblewhite *et al*., 2008; Johnston *et al*., 2009) by up to 10% for a difference in SOM content from 7% to 3% (Gregory *et al*., 2009). Arable soils of low SOM content are susceptible to slumping when wetted due to aggregate instability, leading to less infiltration and greater surface runoff (Whitmore *et al*., 2004).

The quantity and quality of SOM, as the primary food source, largely controls the soil microbial biomass (Fierer *et al*., 2009) and biodiversity (Orwin *et al*., 2006; Wardle *et al*., 2006). Loss of SOM can reduce the exchange of important nutrients such as N, P and S (Johnston *et al*., 2009). SOM loss from 5% to 2% over a 60‐yr period at Rothamsted resulted in a 90% decrease in microbial biomass, but no significant effect on microbial diversity (Hirsch *et al*., 2009) or substrate utilization (Wu *et al*., 2012). Smaller fungal biomass (Gregory *et al*., 2009) and fungal‐to‐bacterial biomass ratios (Bardgett *et al*., 1996, 2007) have been found in soils of low SOM content compared with undisturbed and botanically rich grassland soils in the UK.

Soil organic matter also plays a key role in the ability of soils to buffer the effects of potentially toxic substances, in part by chelation and adsorption (Chander & Brookes, 1993; Hund‐Rinke & Kördel, 2003; Griffiths *et al*., 2005, 2008; Kuan *et al*., 2007). Loss of SOM can, hence, release toxic elements (ROTAP, 2009).

#### Soil contamination

Soil contamination may result from point or diffuse sources. Impacts of the latter are hard to predict as it can be affected by factors such as the weather, and soils far away from the source may be affected. Contamination mainly affects soil biological and chemical properties, although some contaminants (e.g. salts) may destabilize soil structure and affect soil physical properties.

Contamination that decreases soil pH, such as N deposition (NEGTAP, 2001; ROTAP, 2009), reduces nutrient availability, even at moderate levels (pH 5–7), and increases the risk of nutrient leaching (Pearson & Stewart, 1993; Jefferies & Maron, 1997; Degryse *et al*., 2007). Soil pH is one of the main determinants of soil biodiversity and functioning (Fierer & Jackson, 2006; Smolders *et al*., 2009; Griffiths *et al*., 2011). Some keystone soil taxa including *Lumbricidae* (earthworms), *Collembola* (springtails) and N‐fixing bacteria are particularly sensitive to metals (Emmett *et al*., 2010). Contamination can affect the health of soil invertebrates in general (Spurgeon & Hopkin, 1996). Addition of Cu to UK soils (equivalent to 500 mg/kg) decreased both microbial respiration rates by up to 80% initially, and microbial biomass itself (Griffiths *et al*., 2001; Kuan *et al*., 2007; Gregory *et al*., 2009). The effects may not be linear (Hirsch *et al*., 1993; Giller *et al*., 2009) as different groups dominate at different points as toxicity changes. Additions of Cu and Zn increase the metabolic quotient (Rost *et al*., 2001) which is an indicator of microbial stress (Chander & Joergensen, 2001; Chander *et al*., 2002). Metals added in association with sewage sludge may be more toxic than metals added as inorganic salts (Chaudri *et al*., 2008). As a result, contamination can decrease OM mineralization (Giller *et al*., 1998; Dai *et al*., 2004; Emmett *et al*., 2010), N fixation (McGrath, 1998; Broos *et al*., 2004) and the catabolic diversity of soils, measured by the soil's ability to degrade a range of compounds (Wenderoth & Reber, 1999; Girvan *et al*., 2005). Urban soils often have less biodiversity (Fountain & Hopkin, 2004; Styers *et al*., 2010), presumably due to contamination.

#### Soil compaction

Compaction is mainly associated with trafficking in arable soils and livestock in grassland soils. Whereas clay soils are better able to recover, sandy soils are particularly vulnerable (Gregory *et al*., 2009). Subsoil compaction is particularly insidious because it is rarely detected. Recent reviews have summarized comprehensively the causes, effects (some of which are discussed below) and management of soil compaction in the UK (Clarke *et al*., 2007; Batey, 2009).

Soil compaction degrades soil physical quality (Dexter, 1988; Whitmore & Whalley, 2009). Increases in bulk density (of up to 0.18 Mg/m, Ball *et al*., 2008) and soil strength (of up to 3 MPa, Gregory *et al*., 2007; Whalley *et al*., 2008), and decreases in friability (by up to 50%, Watts & Dexter, 1998), soil porosity (by 10–25%, Mooney & Nipattasuk, 2003; Gregory *et al*., 2009; Matthews *et al*., 2010) and water‐holding capacity (Gregory *et al*., 2009) have been reported in UK soils. Compaction preferentially affects macropores (Breland & Hansen, 1996; Richard *et al*., 2001) and can change the alignment of pores from vertical to parallel with respect to the soil surface (Servadio *et al*., 2005). This affects infiltration rates (Heathwaite *et al*., 1990; Kibblewhite *et al*., 2008; Batey, 2009) and hence increases the risk of overland flow, flooding and erosion (Dexter, 1988). Decreases in the saturated hydraulic conductivity of soil by up to three orders of magnitude have been reported (Mooney & Nipattasuk, 2003; Matthews *et al*., 2010). Compaction in grassland soils is less severe than in arable soil (Palmer & Smith, 2013), although it is much less understood (Bilotta *et al*., 2007).

Compaction may have little effect on micro‐organisms as they inhabit small pores which are less affected (Breland & Hansen, 1996; Jensen *et al*., 1996; Kohler *et al*., 2005; Shestak & Busse, 2005; Gregory *et al*., 2007). However, microbial functions may be affected. Breland & Hansen (1996) reported reductions of up to 18% in N mineralization with compaction, ascribed to increased physical protection of SOM and microbial biomass in the smaller pores inaccessible to grazing nematodes created by compaction. Denitrification and NH_3_ volatilization may increase with compaction, causing N loss (Batey & Killham, 1986). Ball *et al*. (2008) found N_2_O emissions increased by up to 4 mg N/m^2^/h following compaction – twice that of the uncompacted control. In contrast, compaction may adversely affect soil macro‐organisms. Reductions in *Collembola* (Heisler & Kaiser, 1995; Schrader & Lingnau, 1997), *Arthropoda* (arthropods) (Aritajat *et al*., 1977a,b) and *Enchytraeidae* (potworms) (Schrader *et al*., 1997; Rohrig *et al*., 1998), and both surface‐dwelling (Piearce, 1984; Radford *et al*., 2001) and deep‐burrowing *Lumbricidae* species (Rushton, 1986; Kretzschmar, 1991) have been reported.

#### Soil sealing

Soil sealing is associated with the main urban areas of the UK under the greatest pressure for housing and infrastructure. In 2011, 82% of the population of England lived in urban areas (ONS, 2012, 2013). Whilst there is little direct evidence of the impacts on soil properties in the UK, general impacts from a European perspective have been discussed (e.g. Huber *et al*., 2008; Scalenghe & Marsan, 2009; Virto *et al*., 2015). Sealing interrupts or removes completely the contact between the soil and other system components such as the biosphere, atmosphere and hydrosphere (Huber *et al*., 2008) with significant and irreversible impacts on the ability of soil to transmit water and gas (Virto *et al*., 2015). Declines in SOM (Wei *et al*., 2014) and microbial biomass and respiration (Piotrowska‐Dlugosz & Charzyński, 2015) have also been reported.

#### Brownfield development

The impact from brownfield development on soil properties is wide‐ranging as determined by the specific prior engineering project. In the extreme case where brownfield development results in a nongreen after‐use, the soil is removed permanently and hence all soil functions will be lost. However, common brownfield developments in the UK include the restoration to a green after‐use of mineral workings, landfill sites and underground infrastructure networks. In these cases, how the soil was removed, stored and reinstated prior to, during and following the lifetime of the industrial use will have a large impact on the success of the green after‐use and the properties and functions of the soil.

The removal and storage of soil often has deleterious effects on soil physical properties, including compaction and loss of structure (HMG, 1996). Soil structure can be damaged through low porosity, up to 15% smaller aggregate stability (Malik & Scullion, 1998), and domination of smaller (<2 mm) aggregates (Edgerton *et al*., 1995; Gregory & Vickers, 2003, 2007). The nutrient status of the soil may be reduced due to leaching during storage and waterlogging if compacted (HMG, 1996). SOM can also be lost (Johnson *et al*., 1988; Bentham *et al*., 1992; Malik & Scullion, 1998; Wick *et al*., 2009). These effects can persist when the soil is reinstated.

The high compaction and shearing forces imparted during lifting, moving and replacement of soil have immediate and significant effects on the biological community (Harris *et al*., 1989, 1993). The soil microbial biomass can decrease by 50% immediately upon removal (Harris *et al*., 1989) and by 95% following storage if conditions become anaerobic (Harris & Birch, 1990). *Lumbricidae* are severely affected, and their populations may take decades to recover even after the soil is reinstated. Fungi and complex organisms are disproportionately affected by engineering operations, because of their physical, multicellular structure. The observed slow recovery of microbial biomass in brownfield soils is linked to the reduced soil physical quality (Edgerton *et al*., 1995; Harris, 2003).

#### Other degradation threats

Salinization, the excess of water‐soluble salts in soils by natural or human‐enhanced means (Huber *et al*., 2008), is limited to those UK soils most susceptible to seawater flooding, or naturally occurring localized acid sulphate soils such as in East Anglia (Dent, 1985; Kibblewhite *et al*., 2008). Salts have a deleterious effect on soil structure and often result in a release of pollutants (Du Laing *et al*., 2009). Though more commonly associated with Mediterranean areas in Europe, particularly mountainous areas (e.g. Ferrara *et al*., 2015; Virto *et al*., 2015), landslides do occur in localized areas of the UK (Foster *et al*., 2012), including coastal cliffs (Bowman & Take, 2015) and upland areas (Gunn *et al*., 2013; Johnson & Warburton, 2015). The result of landslides is often the loss of the entire soil material and hence all properties and functions from a site. As vulnerable sites tend to be in the uplands or by the coast, there can be a significant impact on amenity and agricultural uses (Bowman & Take, 2015). Correspondingly, the soil at the deposition site is buried, severely impairing its functioning.

## Discussion

### The impacts of degradation threats on soil properties in the UK

From the review above, we see that the main degradation threats to soils in the UK affect a wide range of properties within the three main types: physical, biological and chemical. Soil erosion results in the removal of important topsoil and any contained nutrients, C and porosity. A decline in SOM principally affects soil biological and microbiological properties, SOM being the primary food source, but also impacts on soil physical properties because of the effect of SOM on soil structure development and stabilization. Soil contamination affects soil chemical properties, affecting nutrient availability and degrading microbial properties, whilst soil compaction degrades soil physical properties, especially soil porosity. Soil sealing removes the link between the soil and most of the ‘spheres’ and can significantly affect soil hydrological and microbial functions, but may not necessarily affect other soil properties. By contrast, soils on re‐developed brownfield sites typically have degraded physical, chemical and biological properties. From our review, it is apparent that the potential or actual effect of some degradation threats on soils in the UK is well documented, including soil erosion, decline in SOM, soil contamination and soil compaction. By contrast, the impact of soil sealing and brownfield development on soils in the UK appears to be understood less. These areas might need to be research priorities in the future if the UK population (particularly in urban areas) continues to increase.

The spatial arrangement of degradation threats to soils in the UK is complex and beyond the scope of this review. However, we may offer some initial simplistic thoughts. Erosion is associated with overland flow on wet soils or wind in dry soils. Therefore, in the UK, upland soils in the north and west that experience considerable rainfall and arable soils of low porosity may be more prone to suffer from soil erosion by water, whereas light‐textured arable soils may be more prone to wind erosion, such as those in the south and east of the UK. Intuitively, soils with a greater amount of SOM have more potential to lose SOM than soils of lesser SOM. As SOM is to a large extent dictated by land use, soils under grassland, semi‐natural or natural vegetation may be candidates to experience a loss of SOM, particularly if the input from vegetation changes in some way. Grassland dominates soils in the west and north of the UK, whilst the main areas of semi‐natural or natural vegetation are in the uplands. However, it is important to reiterate that there is currently no definitive evidence for significant changes to SOM levels in UK soils (Bellamy *et al*., 2005; Chapman *et al*., 2013; Reynolds *et al*., 2013; Gregory *et al*., 2014). Although compaction is often perceived to be a degradation associated exclusively with trafficking of arable soils, grassland soils can become compacted from the impact of livestock, and hence, compaction can potentially affect many agricultural soils in the UK. The impacts are likely to be more severe in tramlines and where livestock congregate (e.g. feeding and watering troughs) in arable and grassland systems, respectively. Soil sealing and brownfield development are primarily urban‐based degradations and hence will mainly threaten soils within and surrounding the main urban areas in the UK, particularly in the cities in England. Soil contamination could also affect the same soils close to urban and industrial areas, but other contaminants, particular airborne ones, could diffuse to affect soils distant from the source. Continued development and exploitation of soil and land‐use spatial databases (e.g. Mackney *et al*., 1983; Proctor *et al*., 1998; Morton *et al*., 2011) should help to identify soils at risk from particular degradation threats. This kind of approach can then be used to review current land management practices in order to minimize degradation impacts.

### Implications for soil functions in the UK

Having synthesized the literature on the impact of degradation threats on soil properties in the UK, we may then discuss the likely impacts on important soil functions – the tasks or services we ask of soil. These functions typically include food and fibre production, storage of water and C, water and air filtration, support for infrastructure, and support for biodiversity, habitats, cultural and archaeological heritage (HMG, 2011b). We discuss briefly some potential impacts of changes in soil properties arising from degradation threats on soil functions.

Loss of topsoil affects crop yields. A review of 24 studies in the UK found that yields decreased by 4% per 10 cm depth of soil loss, equivalent to a soil loss of around 100 Mg/ha (Bakker *et al*., 2004). Erosion on lowland grasslands, which ranges from 0.5 to 1.2 Mg/ha, can be important locally for growth and silage quality (Bilotta *et al*., 2010). Erosion removes soil habitat space, thus impacting on biodiversity support and water storage functions. Amenity use is also affected by erosion (Rodway‐Dyer & Walling, 2010).

Degradation of soil physical properties, particularly the decrease in porosity from compaction and the destabilization of structure from loss of SOM, affects a range of soil functions. Water storage and flood regulation functions are degraded with a loss in porosity (Dexter, 1997; Whitmore *et al*., 2004; Pilgrim *et al*., 2010). Plant growth can decline as a result of compaction‐imposed loss of porosity, particularly if a dense plough pan develops (van den Akker, 1997). Compaction impairs root penetration (Batey, 2009) and root development (Whalley *et al*., 1995). Specific declines in crop yield have been reported in the UK (Douglas *et al*., 1992; Gregory *et al*., 2007), which can be up to 3 Mg/ha for each 0.1 Mg/m increase in bulk density (Whalley *et al*., 1995) or each 1 MPa increase in strength (Whalley *et al*., 2006, 2008). Gregory *et al*. (2007) reported yield declines of up to 3 Mg/ha in a heavily compacted loamy soil. Compaction can also increase the susceptibility of a crop to soilborne diseases and fungi (Batey, 2009). In arable systems, however, some compaction is beneficial to achieve good seed‐soil contact (Dexter, 1988) and to lessen the risk of lodging (Scott *et al*., 2005).

A decline in soil physical quality has implications for biodiversity support. Plant species differ in their ability to grow in compacted soil (Godefroid & Koedam, 2004a,b), and compacted grassland soils may be less biodiverse than uncompacted soils (Roovers *et al*., 2004). Some evidence exists as to a link between compacted soil and a reduction in soil‐pupating larvae of *Lepidoptera* (butterflies and moths) (Roach & Campbell, 1983). Gilroy *et al*. (2008) reported a negative correlation between soil strength and the abundance of *Motacilla flava* (yellow wagtail) in eastern England, perhaps related to the effect on soil‐dwelling prey. Other British grassland species, including *Turdus philomelos* (song thrush), *Sturnus vulgaris* (starling) and some waders, may be similarly affected (Clarke *et al*., 2007). The quality of soil‐turf systems for amenity is impaired by compaction and trafficking (Marjamaki & Pietola, 2007; Han *et al*., 2008), although compaction is desired in special cases, such as cricket pitches (Baker *et al*., 1998). Compaction may affect the preservation of cultural artefacts and archaeology in the soil (Blum, 1993).

Although the quantitative evidence for critical thresholds is slight (Korschens *et al*., 1998; Loveland & Webb, 2003; Reynolds *et al*., 2007), there is a widely held belief that soil cannot function optimally without an adequate level of SOM (van Camp *et al*., 2004). Often, declines in SOM and structural development are hard to separate. Robinson & Woodun (2008) reported an inverse relationship between SOM content and surface runoff due to crusting in agricultural soils on chalk in southern England. Declines in SOM have been associated with increased erosion (Fullen, 1991) and clay dispersion (Watts & Dexter, 1998). Other functions can also be affected. Correlations have been made between SOM and the abundance of *Diptera* (flies) and aerially active *Coleoptera* (beetles) in arable soils in England (Gilroy *et al*., 2008) and *Culicoides impunctatus* (highland midge) in Scotland (Blackwell *et al*., 1999). A link between SOM loss and the release of toxic elements in soils has also been found (ROTAP, 2009).

Changes in soil chemical properties, chiefly pH arising from agricultural inputs or contaminants, affect many functions. Metals and metalloids can accumulate in topsoil from either the atmosphere or the use of fertilizers, agrochemicals, manures or waste materials on land which, if released in toxic concentrations, particularly under acidifying conditions, can severely impair plant growth and food quality (Pearson & Stewart, 1993; Blake *et al*., 1994; Blake & Goulding, 2002; Millennium Ecosystem Assessment, 2005; Degryse *et al*., 2007; Atkinson *et al*., 2012). Some metals and metalloids are phytotoxic and decrease yields at high concentrations (e.g. Zn, Cu, Cr and As), whereas ‘passage poisons’ affect animal and humans consuming food with little effect on yields (e.g. Cd, Mo, Se and possibly Co) (McGrath & Zhao, 2015). The UK has substantial areas that are contaminated with As (Appleton *et al*., 2012). Acidification can cause plant nutrient imbalances (Phoenix *et al*., 2004), increased risk of nutrient leaching (Pearson & Stewart, 1993; Jefferies & Maron, 1997; Degryse *et al*., 2007) and increased susceptibility of plants to stressors, including diseases and pests (Power *et al*., 1998; Carroll *et al*., 1999). Acid grassland can be toxic to grazing. Above‐ground biomass may be significantly reduced near urban areas due, in part, to the proximity of contaminant sources (Ander *et al*., 2013). Ground‐level O_3_ may also potentially reduce grass (Gonzalez *et al*., 1999) and arable crop yields by up to 15% in the UK, in part by the effect on the ability of plants to respond to drought and to sequester C (ROTAP, 2009). Excess salt levels are toxic to most plant species, but the resulting halophytic ecosystems are often of great biodiversity value however (e.g. salt marsh, Watts *et al*., 2003). Indeed, soil pH is one of the main determinants of plant biodiversity (Bunemann *et al*., 2006; Cookson *et al*., 2006; Rousk *et al*., 2009).

At a fundamental level, where soil sealing removes the link between soils and the other ‘spheres’, functions such as supporting vegetation growth, C sequestration, filtering water and air and supporting biodiversity are significantly or irreversibly reduced (Huber *et al*., 2008; Scalenghe & Marsan, 2009). Also, the degradations in soil properties arising from brownfield development cause problems for re‐creating functioning soils. Restoration to a desired postoperation land use, such as species‐rich grassland, may require intervention (Carrington & Diaz, 2011). However, modern restoration techniques can successfully recreate habitats of high nature conservation value (Tarrant *et al*., 2013), and indeed, soils with poor structure or nutrient status may help in this regard (HMG, 1996).

It is therefore apparent that if potential threats from soil degradation are realized, a range of important soil functions may be affected, through effects on measured soil properties. The studies reviewed above largely focus on functions of immediate current economic value (e.g. crop yields and water resource management) and were often empirical observations rather than mechanistic studies linked to soil properties. Extrapolation of knowledge relating soil properties and soil functions is challenging as a result (HMG, 2011b), even though similar processes may be at work. An associated issue is the potential or otherwise for soils to be truly multifunctional. There are obvious trade‐offs: agricultural monoculture might be at odds with supporting biodiversity, for instance. In the future, a more mechanistic understanding of the response of soil functions to degradation processes, through measured changes in soil properties, would help in this regard.

It is important to note that degradation processes do not necessarily have irreversible effects on soil functions. Land management practices can be revised to reduce or even remove the degradation threat to improve soil properties and functions. Examples in agriculture include reducing compaction by cultivating soil in a brittle rather than plastic state as well as lowering tyre pressures on machinery (Batey, 2009) and decreasing erosion by tilling perpendicular to (rather than up‐and‐down) the slope (Quinton & Catt, 2004). Farmers may also offset SOM loss by incorporating residues or adding organic materials. On nonagricultural land, degradation threats such as contamination, sealing and brownfield site development can probably only be mitigated by policy.

### The effects of climate change on soils in the UK

It is predicted that the UK will experience hotter, drier summers and warmer, wetter winters than currently, with an increased occurrence of low‐frequency‐high‐magnitude events such as heat waves and intense storms (HMG, 2009a,b; Lowe *et al*., 2009). Climate change is likely to affect all the degradation threats, and hence soil properties. We briefly summarize some important implications with reference to observations in the literature and modelling. Whilst it may be possible to look at evidence from regions currently experiencing the kind of climate predicted for the UK, such soils have likely developed over millennia under stable conditions and hence may not necessarily be analogous to how UK soils will respond to rapid climate change.

Soils on the margins of stability are likely to be most vulnerable to drier summers, such as those in eastern England that already experience low rainfall, or peat soils that can suffer irreversible shrinkage upon drying. Prolonged drying can increase soil hydrophobicity (McHale *et al*., 2005) and strength (Whitmore & Whalley, 2009). Rapid drying can increase the mobility of cationic metals (Simpson *et al*., 2010), which can reduce the buffering capacity (Park *et al*., 2010). The likely effect on SOM is unknown: warming may increase microbial activity (Cox *et al*., 2002; Carney *et al*., 2007) or net primary productivity (Smith *et al*., 2007; Johnston *et al*., 2009), resulting in either a net loss or gain in soil C, respectively. Some models predict significant loss of soil C across much of the UK up to 2080 (Smith *et al*., 2005). Suseela & Dukes (2013) suggested a seasonality effect on SOM with decreased respiration during the growing season and increased respiration in the nongrowing season. Global warming is predicted to cause a sea‐level rise of between 3 and 9 cm between 2010 and 2030 (Lowe *et al*., 2009), which will increase the risk of flooding and salinization in low‐lying coastal soils of the UK.

The wet winter experienced in the UK in 2000–2001 provided some insight into future rainfall patterns (ADAS, 2002). Soils suffered from prolonged waterlogging, rill erosion, losses of NO_3_ (in drainage water), S (in SO_4_) and P (in sediment), and precipitation of Fe and Mn (ADAS, 2002). Waterlogging causes anaerobism and has been found to reduce N, P and K nutrient uptake rates to <10% of control soils and to increase the concentration of potentially harmful compounds including C_2_H_4_ (up to 6 *μ*L/L) and Mn (up to 0.5 mg/kg) (Drew & Sisworo, 1979). McTiernan *et al*. (2001) reported 70–300% greater losses of dissolved organic C from grassland that was seasonally waterlogged rather than well drained, although Scholefield *et al*. (1993) found increased NO_3_ leaching from drained soil at the same site. Unger *et al*. (2009) found that flooding reduced total microbial biomass, Gram‐positive bacteria and mycorrhizal fungi by up to 50%. Milan (2012) reported lasting effects of high‐magnitude flooding in an upland catchment in the UK where soil adjacent to the river was lost together with its vegetation. The overall increase in flood frequency in the UK may be as great as 50% at the current 50‐yr return period in some catchments (Kay *et al*., 2006). The full effects of significant flooding in southern England in the winter of 2013–2014 have yet to be assessed.

Changes in soil properties arising from climate change will affect important soil functions, particularly food and fibre yields. With increased drying, rainfed agriculture could become risky for some important crops in the UK such as potato (Daccache *et al*., 2012). Following the wet winter in the UK in 2000–2001, yields of winter wheat (12% decline), barley (7.5% decline), spring oilseed rape crop, sugar beet and potato were significantly reduced (ADAS, 2002). Rounsevell & Brignall (1994) concluded that machinery working days would be reduced should autumnal precipitation increase in England by 15%, which is entirely possible. Flooding makes it difficult to get livestock onto the land (Tyson *et al*., 1992), and animals grazing floodplains are at risk of being lost in floodwater (Blackwell & Maltby, 2006). While the greater atmospheric CO_2_ may enhance yields of temperate plants (Long *et al*., 2004), it could come at a cost of reduced plant protein content (Cotrufo *et al*., 1998; DaMatta *et al*., 2010), if N becomes limited in the soil (Gill *et al*., 2002). Further impacts on soil functions may be apparent. Increased concentrations of CO_2_ may reduce plant transpiration (Körner, 2000), which could reduce the ability of the soil to store additional rainfall. Substantially reduced flows in wetlands may reduce their water regulatory capacity (Sutherland *et al*., 2008). Waterlogging causes reducing conditions in soils, which could impact on buffering and contaminant filtering (Du Laing *et al*., 2009; Lair *et al*., 2009). The impact of climate change on soil biodiversity remains unclear, although it will reflect changes in general soil conditions and vegetative growth. Resilience in biodiverse habitats at the larger scale may mask changes at fine scales (Fridley *et al*., 2011). A link between climate, shrink–swell cycles in clay soils and subsidence claims from properties damaged in the UK has been reported (Harrison *et al*., 2012), and it is predicted that the magnitude and frequency of landslides in the UK will increase in the future (Pritchard *et al*., 2014). There is conflicting evidence as to whether waterlogging and anaerobism will help to preserve (Caple, 1996; Raiswell, 2001) or degrade (Douterelo *et al*., 2010) archaeological materials in soils.

It would appear that soils will need to be carefully managed in the future to minimize any exacerbating effects of climate change on the range of degradations that they currently experience.

## Conclusions

With the UK as an example, we have summarized some key data on the impact of degradation threats on soil properties and have sought to link changes in soil properties to important soil functions. The key next stage will be to continue to add to the database with field‐based monitoring of broad changes to soil properties following degradation supported by controlled laboratory experimentation to improve understanding of the mechanisms involved. Outputs from such an approach, together with refinement of soil and land‐use spatial databases, should aid the development of models capable of extrapolating up to the national scale in order to link soil degradations to soil functions. We may also be able to predict any effect that climate change is likely to have. Armed with this, we may revise our policies so that soils are truly managed sustainably.
